# Butylated Hydroxytoluene Induced Resistance Against *Botryosphaeria dothidea* in Apple Fruit

**DOI:** 10.3389/fmicb.2020.599062

**Published:** 2021-01-14

**Authors:** Yan Huang, Cuicui Sun, Xiangnan Guan, Sen Lian, Baohua Li, Caixia Wang

**Affiliations:** ^1^Key Laboratory of Integrated Crop Pest Management of Shandong Province, College of Plant Health and Medicine, Qingdao Agricultural University, Qingdao, China; ^2^Knight Cancer Institute, Oregon Health & Science University, Portland, OR, United States

**Keywords:** butylated hydroxytoluene, apple fruit, induced resistance, *Botryosphaeria dothidea*, defense-related enzyme, salicylic acid signaling

## Abstract

Apple ring rot caused by *Botryosphaeria dothidea* is an important disease in China, which leads to serious economic losses during storage. Plant activators are compounds that induce resistance against pathogen infection and are considered as a promising alternative strategy to traditional chemical treatment. In the present study, butylated hydroxytoluene (BHT), a potential plant activator, was evaluated for its induced resistance against *B. dothidea* in postharvest apple fruits. The physiological and molecular mechanisms involved in induced resistance were also explored. The results showed that BHT treatment could trigger strong resistance in apple fruits against *B. dothidea*, and the optimum concentration was 200 μmol L^–1^ by immersion of fruits. BHT treatment significantly increased the activities of four defensive enzymes and alleviated lipid peroxidation by increasing antioxidant enzyme activities. In addition, salicylic acid (SA) content was enhanced by BHT treatment as well as the expression of three SA biosynthesis-related genes (*MdSID2*, *MdPAD4*, and *MdEDS1*) and two defense genes (*MdPR1* and *MdPR5*). Our results suggest that BHT-conferred resistance against *B. dothidea* might be mainly through increasing the activities of defense-related enzymes and activating SA signaling pathway, which may provide an alternative strategy to control apple ring rot in postharvest fruits.

## Introduction

Apple (*Malus domestica* Borkh.) is one of the most important fruit and is widely consumed worldwide. However, the apple fruit is susceptible to pathogenic fungi, one of which is *Botryosphaeria dothidea* (Tang et al., 2012). *B. dothidea* is a plant hemi-biotrophic fungi and causes apple ring rot, a severe disease that reduces the yield and quality of apple, especially in China. Infected fruits show soft, sunken lesions with alternating tan and brown rings. *B. dothidea* infects fruits at the growth stage and causes fruit rot during the ripening or in the postharvest period (Zhang Q. M. et al., 2016), making it difficult to control apple ring rot. In addition, *B. dothidea* also infects apple tree branches, resulting in warts, canker, necrotic bark lesions, and even the death of the branches (Zhao et al., 2016).

Currently, the main strategy to control apple ring rot is the eradication of the fungal inoculum combined with application of bagging technology and scheduled spraying of protective fungicides (Zhao et al., 2016). As the cost of bagging is increasing yearly, the baggless cultivation of apple has become an inevitable trend in China. However, apple fruit ring rot is the key problem that remains to be solved in baggless cultivation (Zhao et al., 2016; Yu et al., 2018). Furthermore, issues associated with chemical residue, fungicide resistance in pathogens, and serious environmental pollution have motivated to develop new, effective, and eco-friendly agents for the control of apple ring rot (Fan et al., 2016, 2018).

Plant activators that induce plant defense response have attracted considerable interest due to their broad-spectrum resistance and long-term disease control while reducing the environmental burden (Yang et al., 2015; Liu et al., 2019). As an alternative strategy to traditional chemical treatment, the application of activators has gained increasing attention (Yu et al., 2014; Zhang Y. et al., 2016). The plant activators include physical, biological, and chemical inducers, while most are compounds. Complex signaling networks, such as the pathways mediated by salicylic acid (SA), jasmonic acid (JA), and ethylene (ET), are activated by plant activators, leading to plant defense response (Van Bockhaven et al., 2015; Yu et al., 2019). SA-mediated signaling plays important roles in fighting against (hemi)-biotrophic pathogens (Glazebrook, 2005) as well as in the activation of robust systemic resistance marked by the increased expression of many defense proteins including pathogenesis-related (PR) proteins. In contrast, JA- and/or ET-mediated signaling is mainly involved in plant defense against necrotrophic pathogens.

As metabolic by-products in plants, reactive oxygen species (ROS) play a pivotal role in the regulation of plant defense response against pathogen invasion (Torres, 2010; Wang et al., 2014). However, ROS produced in excess can cause oxidative damage and induce membrane lipid peroxidation in the cellular environment (Marschall and Tudzynski, 2016). To protect cells from oxidative damage, plants employ a complex ROS–antioxidative system to scavenge harmful ROS. The main scavenging mechanism includes enzymatic antioxidants and metabolites that facilitate their degradation (Mittler, 2002).

In the previous work, we demonstrated that *Streptomyces rochei* A-1 could induce resistance against *B. dothidea* infection in postharvest apple fruit and did not affect the external and internal fruit appearance (Zhang Q. M. et al., 2016). It was recently found that butylated hydroxytoluene (BHT) was one of the main metabolites of *S. rochei* A-1 and that low concentration of BHT could effectively inhibit the postharvest apple fruit decay caused by *B. dothidea*. However, low concentration of BHT has no effect on mycelial growth and spore germination of *B. dothidea* (Yu et al., 2018), suggesting other possible mechanisms of BHT in inhibiting postharvest decay in apple fruits. Moreover, BHT could inhibit postharvest gray mold caused by *Botrytis cinerea* and bitter rot caused by *Colletotrichum gloeosporioides* (data not published). As a synthetic phenolic antioxidant, BHT has been widely used in foods and food-related products, such as packing (Babich, 1982). Furthermore, BHT could activate several antioxidases and regulate endogenous nitric oxide against abiotic stress conditions in *Haematococcus pluvialis* (Zhao et al., 2018).

In this study, we set out to investigate the effectiveness of BHT on the control of apple ring rot caused by *B. dothidea* in postharvest apple fruit. The possible mechanisms of action underlying BHT-induced resistance to *B. dothidea* and the association with the SA signaling pathway in apple fruits were also explored. Our results suggested BHT treatment as a promising strategy to protect fruits from pathogen infection.

## Materials and Methods

### Butylated Hydroxytoluene, Fungal Pathogen, and Fruit Materials

Butylated hydroxytoluene was purchased from Sigma-Aldrich (St. Louis, United States). The stock solution (1 M in methanol) was filtered through a 0.22-μm filter for sterilization and stored at −20°C. The working concentration of BHT in this study was 0 (0.1% methanol as control), 50, 100, 200, and 500 μmol L^–1^.

In this study, the fungal pathogen *Botryosphaeria dothidea* LXS030101, which was used in our previous study (Zhang Q. M. et al., 2016), was isolated from infected “Red Fuji” fruits with typical apple ring rot symptoms. The pathogen was maintained on potato dextrose agar (PDA: the extract of 200 g of potato, 20 g of glucose, and 15 g of agar in 1 L of water) at 4°C and was revived on PDA plates for 3 days to obtain the culture. Conidia were harvested from young apple fruits following the method described by Leng et al. (2009). Spore concentration was determined by a hemocytometer and adjusted with sterile distilled water to the concentration of 1 × 10^5^ spores ml^–1^.

Apple (cv. “Fuji”) fruits were harvested at a commercial mature stage from a commercial orchard in Qingdao, China. For this study, fruits with uniform shape, size, and no physical injuries were selected. Before treatment, fruits were surface disinfected with 2% (v/v) sodium hypochlorite for 2 min, washed with tap water, and then air-dried for use in the experiments.

### Induction of Apple Fruit Resistance Against *Botryosphaeria dothidea* by Butylated Hydroxytoluene

Prior to pathogen inoculation, apple fruits were pre-treated with BHT according to the previous procedure with some modifications (Yang et al., 2017). Briefly, apple fruits were immersed in the BHT solution for 15 min at different concentrations (0, 50, 100, 200, and 500 μmol L^–1^). Then, all fruits were taken out, air-dried for 2 h at room temperature, and stored in plastic boxes. After 48 h of incubation at 25°C, three uniform wounds (5 mm wide and 3 mm deep) around the apple equator were punctured with a sterile borer (Zhang Q. M. et al., 2016). Each wound was inoculated with 10 μl of *B. dothidea* spore suspension at 1 × 10^5^ spores ml^–1^. All treated fruits were incubated at 25°C and high relative humidity of 90–95%. Disease severity was expressed by disease incidence and lesion areas, which were recorded at 3, 5, and 7 days post inoculation (dpi). Disease incidence was calculated as the percentage of infected wounds, and lesion diameter was measured for infected wounds only. The experiment was conducted three times in triplicates with 10 fruits in each replicate group.

### Treatment of Apple Fruit and Sample Collection

Apple fruits were divided into two groups, immersed in 0 μmol L^–1^ (0.1% methanol as control) or 200 μmol L^–1^ of BHT solution, air-dried for 2 h, and then stored at 25°C with high relative humidity. Sample collection was performed following the method of Zheng et al. (2011). Peels from the fruit equator area were excised and cut into small pieces at time points 0, 0.5, 1, 2, 3, 5, and 7 days after treatment. Each treatment included three replicates, and each replicate consisted of five fruits. The excised tissues from five fruits were mixed and encased in aluminum foil, snap frozen in liquid nitrogen, and then stored at −86°C until further biochemical assays and gene expression analysis. All the experiments were repeated thrice.

### Determination of Defensive Enzyme Activities

Phenylalanine ammonia lyase (PAL; EC 4.3.1.5), polyphenol oxidase (PPO; EC 1.10.3.1), β-1,3-glucanase (GLU; EC 3.2.1.58), and chitinase (CHI, EC 3.2.1.14) were assayed according to the method of Zhang Q. M. et al. (2016), Zhang Y. et al. (2016). Fruit tissues (0.5 g) were homogenized with 5 ml of 100 mmol L^–1^ of phosphate-buffered saline (PBS; pH 6.8–8.8) containing 1% (w/v) polyvinylpyrrolidone (PVP) for PPO and PAL, and 50 mmol L^–1^ of sodium acetate buffer (pH 5.0) for GLU and CHI. The homogenate was centrifuged for 15 min at 12,000 *g* at 4°C, and the supernatants were used to determine the enzyme activities. The activities of PAL, PPO, GLU, and CHI were calculated on the basis of fresh weight (FW), which were expressed as U g^–1^ FW. All measurements were performed in triplicate with samples collected from three biological replicates.

### Determination of Antioxidant Enzyme Activities and Lipid Peroxidation

To determine the activities of main antioxidant enzymes including catalase (CAT, EC 1.11.1.6), peroxidase (POD, EC 1.11.1.7), and superoxide dismutase (SOD, EC 1.15.1.1), 0.5 g of fruit tissues was homogenized with 5 ml of 100 mmol L^–1^ PBS (pH 7.0) containing 1% PVP (w/v). The CAT Assay Kit A007, POD Assay Kit A084, and SOD Assay Kit A001 (Nanjing Jiancheng Bioengineering Institute, Nanjing, China) were used to measure the enzyme activities, following the protocol provided by the manufacturers. The final activity was expressed as U g^–1^ FW. Three biological replicates were used in each treatment.

To analyze the lipid peroxidation in apple fruits, four treatment groups were designed as follows: (i) control, fruits treated with 0.1% methanol; (ii) BHT, fruits treated with 200 μM of BHT solution; (iii) pathogen, fruits treated with 0.1% methanol followed by *B. dothidea* inoculation; (iv) BHT + pathogen, fruits treated with 200 μmol L^–1^ of BHT followed by *B. dothidea* inoculation. Fruit tissues were collected at various intervals (0, 1, 2, 3, 4, 5, and 7 dpi) as the description of Zhang Y. et al. (2016). Malondialdehyde (MDA) content was measured to determine lipid peroxidation in fruit tissues with an MDA Assay Kit A003 (Nanjing Jiancheng Bioengineering Institute, Nanjing, China). The MDA content was expressed as mmol per kg of FW. Three replicates were carried out in this experiment.

### Analysis of Gene Expression by Quantitative Real-Time PCR

Spin column plant total RNA purification kit was used to extract total RNA from fruit tissues (Sangon Biotech, Shanghai, China). After quantification by micro spectrophotometry, a total of 2 μg of RNA was used for first-strand cDNA synthesis, using the Prime Script RT Reagent Kit with gDNA Eraser (Takara, Dalian, China).

qRT-PCR was performed using SYBR Premix Ex Taq kit (Takara, Dalian, China) according to the protocol provided by the manufacturer. Primers used for qRT-PCR are listed in Table 1. The qRT-PCR thermal cycling program was as follows: initial denaturation for 30 s at 95°C, 40 cycles of 5 s at 95°C, 15 s at 59°C (or 58°C or 56°C), and 15 s at 72°C. Elongation factor 1-a (*EF1a*) was used as a reference gene, and the relative gene expression was normalized to the *EF1a* Ct value according to the formula 2^–Δ^ ^Δ^ ^*Ct*^. All experiments were carried out with at least three biological replicates.

**TABLE 1:** Specific primers used for quantitative real-time PCR to analyze gene expression.

Primer name	Forward primer 5′→3′	Reverse primer 5′→3′	Annealing temperature (°C)
*MdEF1*α	ACATTGCCCTGTGGAAGTT	GTCTGACCATCCTTGGAAA	59/58/56
*MdPR1*	GCAGCAGTAGGCGTTGGTCCCT	CCAGTGCTCATGGCAAGGTTTT	59
*MdPR5*	AGCAGCTTCCCTCCTCGGC	CCCAGAAGCGACCAGACC	58
*MdPDF1.2*	ATGTTTGGACTGATGTGGCAA	TCAACATCTGAAGTAGCAGAAG	58
*MdCOI1*	CTGACTTCCCTTAGGTACTTGTG	CAACTCGTCTCGGAGGAATCAA	58
*MdERF3*	TCCTTCAAAGCTCCGCTGACTT	CCAAGATGGTGCCTGGAAATCA	58
*MdCTR1*	AACTTGTCCATAGTCACGGAA	CCTTTGCCACATCATATGCC	58
*MdSID2*	TTATACTTCATTCCGCTGCT	GCCTCTAATTTTCTTTGTATGCT	56
*MdEDS1*	GAGCTAGACAATGCCTTCGT	AGTATCCCTCATTGTGCTCGT	58
*MdPAD4*	GCTTCACCGTAAGTTACTCG	CAAGAAACTCGCAACTGTC	58

### Determination of Salicylic Acid Content

Salicylic acid and SA glucoside (SAG) were extracted and measured from 0.3 g of fresh fruit tissue, as described by the previous method with some modifications (Verberne et al., 2002). The sample was homogenized with 1 ml of 90% methanol, and anisic acid was added as an internal standard. The homogenate was centrifuged at 6,000 *g* for 5 min, and the supernatant was collected and concentrated with nitrogen. Trichloroacetic acid was added to a concentration of 1 g L^–1^. The sample was extracted three times with 1:1 ethylacetate:cyclohexane and used to detect the free SA. For SAG detection, 8 mol L^–1^ of HCl was added into the water phase collected from the abovementioned steps. The sample was boiled for 30 min and extracted as the abovementioned method. Samples were analyzed by an Agilent 1260 (Agilent Technologies, Palo Alto, CA, United States). SA and SAG contents were expressed as micrograms per kilogram of FW, and the results were the average of four independent extractions. The experiment was repeated twice.

### Statistical Analysis

All the experiments were implemented using a completely randomized design. The biochemical and gene expression analyses of fruit tissues for each treatment were conducted in triplicate at each sampling point. The data were expressed as the mean ± standard deviation (SD). All data were subjected to analysis of variance (ANOVA) followed by Duncan’s multiple range tests. Statistical significance was determined with a *P* value of less than 0.05.

## Results

### Induction of Disease Resistance in Apple Fruit Against *Botryosphaeria dothidea* by Butylated Hydroxytoluene

**FIGURE 1:**
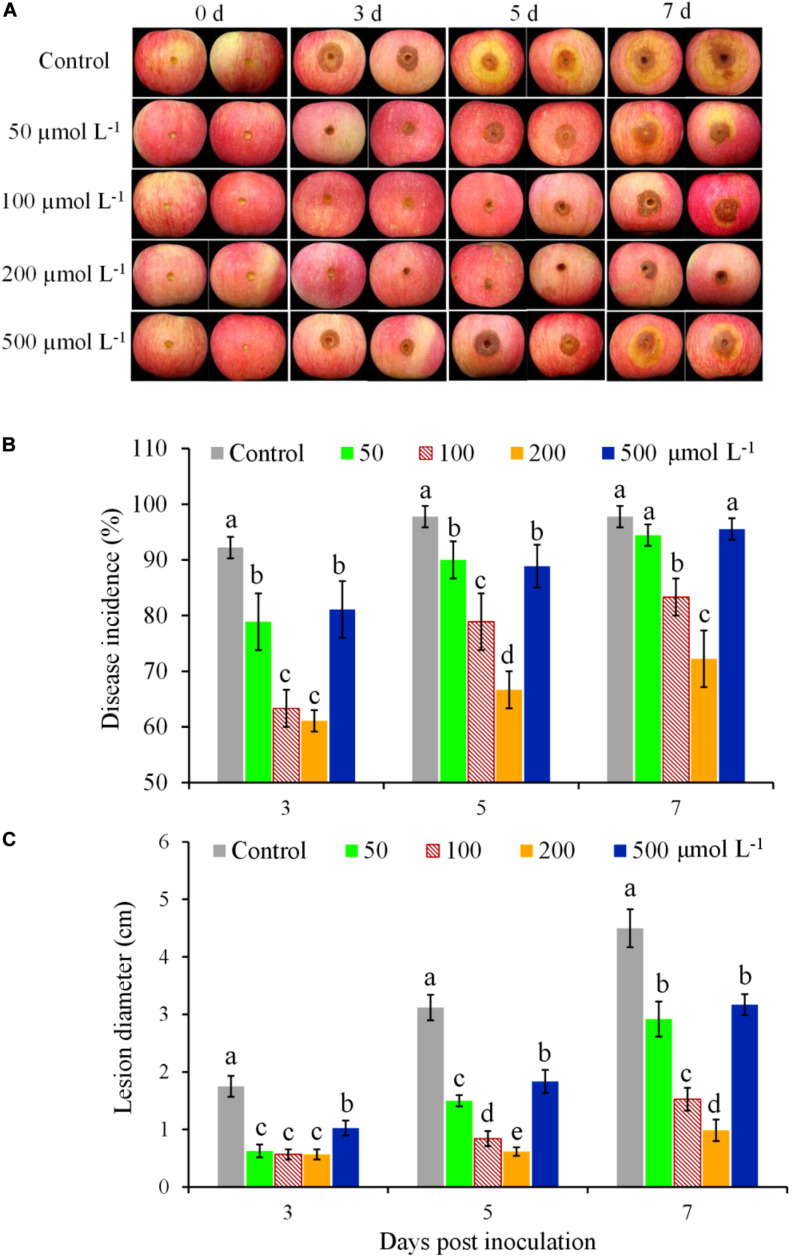
Induced resistance at different butylated hydroxytoluene (BHT) concentrations against *Botryosphaeria dothidea* in apple fruits during postharvest storage. Apple fruits were immersed in 0.1% methanol (as the control) or BHT solution at different concentrations (50, 100, 200, and 500 μmol L^–1^) for 15 min; 48 h after the treatment, all fruits were inoculated with 10 μl of *B. dothidea* conidia suspension (1 × 10^5^ spores ml^–1^) and then stored at 25°C and relative humidity (RH) of 90–95%. Images of representative samples **(A)** are shown for 3, 5, and 7 days post inoculation (dpi). Changes of disease incidence **(B)** and lesion diameter **(C)** in apple fruits were measured at 3, 5, and 7 dpi. Each column represents the mean of three replicates, and vertical bars represent the standard deviation (SD). Different letters above the bars indicate statistically significant differences (*P* < 0.05) within the same panel.

The effectiveness of BHT for controlling apple ring rot in fruits was significantly affected by the concentrations of BHT (Figure 1). In the control fruits treated with 0.1% methanol, brown lesions with large zones appeared on the inoculation site and developed rapidly (Figure 1A). In contrast, the development of ring rot symptoms was significantly inhibited by BHT treatment, as indicated by the lower disease incidence and smaller lesion diameter in BHT-treated fruits compared with control until 7 dpi (Figures 1B,C). Meanwhile, BHT at the concentration of 200 μmol L^–1^ showed the strongest effect on mitigating ring rot development (Figure 1A). Although disease incidence and lesion diameter increased from 3 to 7 dpi in all treatments, 200 μmol L^–1^ of BHT treatment decreased disease incidence by 25.56% and lesion diameter by 78.22% as compared with control at 7 dpi (Figures 1B,C).

### Effect of Butylated Hydroxytoluene on Defensive Enzyme Activities in Apple Fruit

**FIGURE 2:**
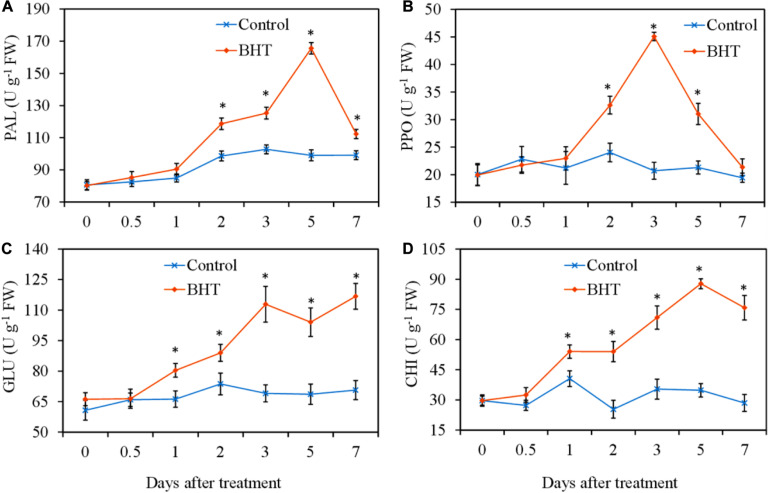
Effects of 200 μmol L^–1^ of butylated hydroxytoluene (BHT) on the activities of phenylalanine ammonia lyase (PAL) **(A)**, polyphenol oxidase (PPO) **(B)**, β-1,3-glucanase (GLU) **(C)**, and chitinase (CHI) **(D)** in apple fruits. The fruits were incubated for various time intervals at 25°C after BHT treatment. Each data point represents the mean ± SD of three replicates. Asterisks denote significant difference from control at a given time point (*P* < 0.05).

To analyze whether BHT treatment was able to increase the defensive enzyme activities, PAL, PPO, GLU, and CHI activities were measured in apple fruits with or without BHT treatment. As shown in Figure 2, four defensive enzyme activities in the control apple fruits exhibited no obvious changes during the storage, whereas the enzyme activities in BHT-treated fruits increased significantly (*P* < 0.05) with varying patterns. PAL and PPO activities in BHT-treated fruits were remarkably enhanced at 2 days and then peaked at 5 and 3 days, which were 1.7-fold and 2.2-fold higher than those in the control fruits, respectively (Figures 2A,B). The activities of PAL and PPO in BHT-treated fruits then dropped sharply. The PPO activity decreased to the control level at 7 days, whereas the PAL activity in BHT-treated fruits was significantly (*P* < 0.05) higher than that in the control fruits during the storage (Figures 2A,B).

For CHI and GLU, BHT-treated fruits exhibited significantly higher enzyme activities from day 1 onward as compared with the control fruits (Figures 2C,D). Particularly, in BHT-treated fruits, GLU activity increased continuously and peaked at 7 days, which was 1.7 times more than that of the control (Figure 2C). Meanwhile, CHI activity reached the highest level at 5 days, which was 2.5-fold higher than that in the control fruits (Figure 2D). These results suggested that BHT could enhance disease resistance against *Botryosphaeria dothidea* by heightening the activities of defensive enzymes PAL, PPO, GLU, and CHI.

### Effect of Butylated Hydroxytoluene on Antioxidant Enzyme Activities and Lipid Peroxidation in Apple Fruit

**FIGURE 3:**
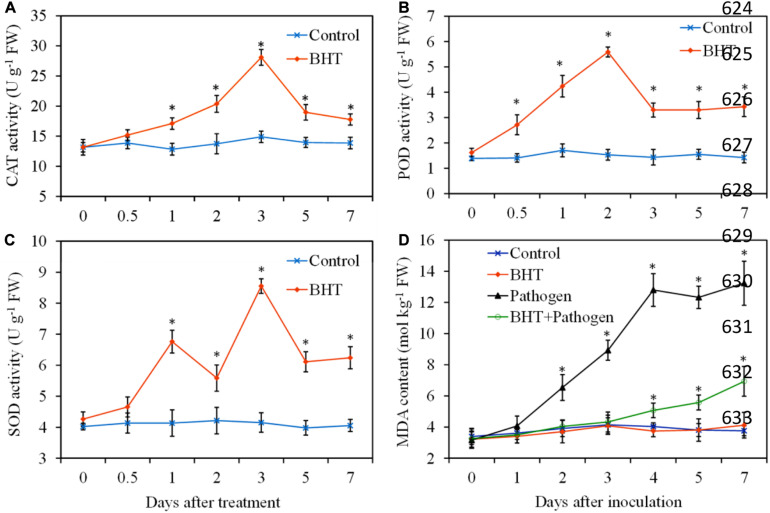
Effects of 200 μmol L^–1^ of butylated hydroxytoluene (BHT) on catalase (CAT) **(A)**, superoxide dismutase (SOD) **(B)**, and peroxidase (POD) **(C)** activities and lipid peroxidation as measured by malondialdehyde (MDA) content **(D)** in apple fruits. The fruits were incubated for various time intervals at 25°C. Each data point represents the mean ± SD of three replicates. Asterisks denote significant difference from control at a given time point (*P* < 0.05).

To obtain more insights into the mechanisms of BHT in induced resistance against *B. dothidea*, antioxidant enzyme activities and lipid peroxidation were determined during apple fruit storage, with or without BHT treatment. In the control fruits, the antioxidant enzymes (CAT, POD, and SOD) activities showed no obvious changes throughout the experiment period; however, three enzyme activities markedly enhanced in BHT-treated fruits (Figures 3A–C). CAT activity increased slowly and reached the maximum levels at 3 days in fruits treated with BHT, which was 2.1-fold higher than that in the control fruits. Similarly, BHT treatment resulted in an evident increase of POD activity, and the maximum fold increase was reached at 2 days (3.6-fold higher than that in the control fruits). For SOD, two activity peaks appeared at 1 and 3 days after BHT treatment. Subsequently, three enzyme activities sharply decreased after the peaks in BHT-treated fruits; however, the enzyme activities remained significantly (*P* < 0.05) higher than those in the control fruits during the storage.

Malondialdehyde, a marker for monitoring lipid peroxidation caused by oxidative damage, was determined in four treatments and shown in Figure 3D. The MDA content in BHT-treated fruits still had no significant (*P* > 0.05) differences compared with that in the control fruits during the storage. Inoculation with *B. dothidea* resulted in a sharp rise of MDA content in apple fruits from 2 dpi, and the increase of MDA content was up to 197.77% and 251.86% more than the control at 4 and 7 dpi, respectively. However, MDA content in BHT plus *B. dothidea*-treated fruits increased slightly, and the enhancement was only 25.81 and 84.30% more than that of the control at 4 and 7 dpi, respectively.

### Effects of Butylated Hydroxytoluene on Relative Expression of Salicylic Acid/Jasmonic Acid/Ethylene Signaling Pathway Marker Genes in Apple Fruit

**FIGURE 4:**
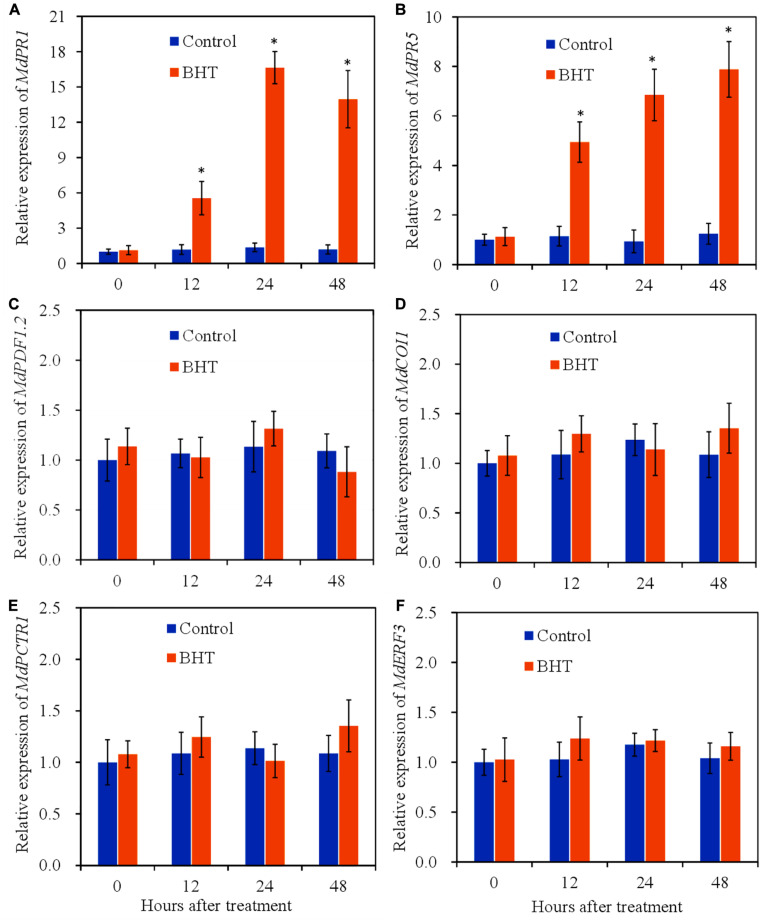
Effects of 200 μmol L^–1^ of butylated hydroxytoluene (BHT) on salicylic acid/jasmonic acid/ethylene (SA/JA/ET) signaling-related gene expressions in apple fruits: **(A)** *MdPR1*, **(B)** *MdPR5*, **(C)** *MdPDF1.2*, **(D)** *MdCOI1*, **(E)** *MdCTR1*, and **(F)** *MdERF3*. The fruits were incubated for various time intervals at 25°C. Each bar represents the mean ± SD of three biological replicates. Asterisks denote significant difference from control at a given time point (*P* < 0.05).

To determine the activation of signaling pathways by BHT, the relative expression levels of marker genes of SA/JA/ET signaling pathway at 0, 12, 24, and 48 h after treatment were analyzed. *PR1* and *PR5* are widely used molecular markers that correlate with SA signaling activation (Zhang Y. et al., 2016). Compared with the control, BHT treatment triggered significant (*P* < 0.05) increases of *MdPR1* and *MdPR5* transcript levels during the storage (Figures 4A,B). The expression of *MdPR1* in BHT-treated fruits showed 4. 7-, 12. 2-, and 11.8-fold increases at 12, 24, and 48 h, respectively, when compared with that in the control fruits (Figure 4A). Meanwhile, a similar pattern was observed in the expression of *MdPR5* in BHT-treated fruits, which were 4. 3-, 7. 4-, and 6.4-fold higher than that in the control fruits.

*PDF1.2* (plant defensin 1.2), which encodes an antifungal peptide, is reported to be induced by pathogen and is required for the activation of JA response pathway (Penninckx et al., 1998). Coronatine insensitive 1 (*COI1*) is a JA receptor responsible for transducing JA signal to activate gene expression in JA signaling pathway (Browse, 2009). In the fruit treated with 0.1% methanol (control), no statistically significant differences (*P* > 0.05) were observed in the two JA signaling pathway marker genes (Figures 4C,D). Meanwhile, BHT treatment also did not induce the significant enhancement of *MdPDF1.2* and *MdCOI1* transcript levels during the storage.

Additionally, constitutive triple response 1 (*CTR1*) and ET response factor 3 (*ERF3*) are used as marker genes of ET signaling pathway (Guo and Ecker, 2004; Vahala et al., 2013). *CTR1* encodes a Raf-like protein kinase and functions as a negative regulator in ET signaling pathway, while *ERF3* is induced in plants exposed to ET. As shown in Figures 4E,F, the expression profile of *CTR1* and *ERF3* exhibited a similar pattern in BHT-treated fruits. BHT treatment could not trigger the up-regulation of the two ET signaling pathway marker genes. These results together suggested that BHT treatment might mainly up-regulate SA signaling pathway.

### Effects of Butylated Hydroxytoluene on Salicylic Acid Content and Salicylic Acid Synthesis-Related Gene Expressions in Apple Fruit

**FIGURE 5:**
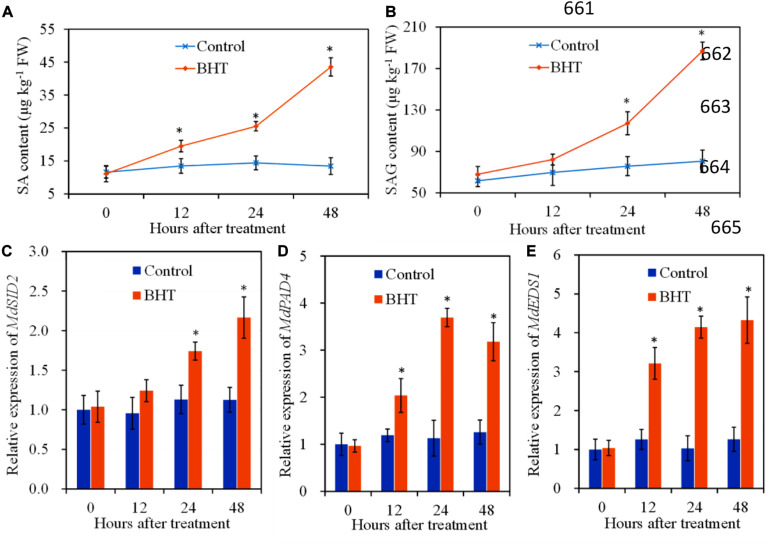
Effects of 200 μmol L^–1^ of butylated hydroxytoluene (BHT) on salicylic acid (SA) contents and SA synthesis-related gene expressions in apple fruits: **(A)** SA content; **(B)** SA glucoside (SAG) content; **(C)** *MdSID2*, **(D)** *MdPAD4*, and **(E)** *MdEDS1*. The fruits were incubated for various time intervals at 25°C. Each bar represents the mean ± SD of four replicates. Asterisks denote significant difference from control (*P* < 0.05) at a given time point.

To investigate the regulation of SA signaling pathway mediated by BHT in apple fruits, SA content and relative expression of three genes related to SA biosynthesis were analyzed, with or without BHT treatment. The free SA content and SAG level changed slightly in the control apple fruits during the storage; however, free SA and SAG contents increased remarkably in BHT-treated fruits at 12 and 24 h, respectively (Figures 5A,B). Free SA and SAG levels continued to accumulate; and at 48 h, the levels were 3.2- and 2.3-fold higher than those in the control fruits, respectively.

*SID2* (SA induction deficient 2), *PAD4* (phytoalexin deficient 4), and *EDS1* (enhanced disease susceptibility 1) are genes related to SA biosynthesis (Dempsey et al., 2011). The relative expression of *MdSID2* in the control fruits exhibited no obvious change during the storage, while the transcript levels of *MdSID2* in BHT-treated fruits increased significantly (*P* < 0.05) at 24 and 48 h (Figure 5C). Similarly, BHT treatment up-regulated the expression of *MdPAD4* and *MdEDS1* during the storage. The transcript levels of *MdPAD4* and *MdEDS1* in BHT-treated fruits reached the highest at 24 and 48 h, which were 3.3- and 3.4-fold higher than those in the control fruits (Figures 5D,E).

## Discussion

As a promising alternative to synthetic chemical fungicides in managing plant diseases, many plant activators have been widely explored for their potential application, especially in controlling postharvest diseases (Worrall et al., 2012; Liu et al., 2019). In the past two decades, there has been growing interest in finding novel plant activators to meet the requirements of plant diseases controlling (Du et al., 2012). BHT is a most commonly used antioxidant in food containing fats, pharmaceuticals, etc. (Yehye et al., 2015). Furthermore, it has been found that BHT is effective as an antioxidant with radical-scavenging activity in living systems (Fujisawa et al., 2004). However, little knowledge was available regarding the effect of BHT applied for postharvest disease control. In the present study, our data showed the excellent efficacy of BHT to control postharvest apple ring rot. Especially, we investigated the mechanism of BHT induced resistance against *Botryosphaeria dothidea* infection and found that SA signaling pathway was associated with BHT-mediated defense response in apple fruits.

To further study BHT-induced resistance in apple fruits against *B. dothidea*, we applied different concentrations of BHT by immersion of apple fruits and evaluated the effects of BHT on disease incidence and disease severity as measured by lesion diameter. As shown in Figure 1, BHT applied by immersion of fruits at 50∼500 μmol L^–1^ could effectively reduce disease incidence and lesion diameter as compared with the control, indicating that BHT played a positive role in apple fruits against *B. dothidea*. However, the inhibitory efficiency of BHT against *B. dothidea* did not depend on the dose. The disease incidence and lesion diameter on the fruits treated with 500 μmol L^–1^ of BHT were significantly higher than those on the fruits treated with 100∼200 μmol L^–1^ of BHT. These results are consistent with previous reports that showed that the high doses of melatonin and L-arginine used on tomato had lower control efficiency against *Botrytis cinerea* than had low-dose compounds (Zheng et al., 2011; Liu et al., 2019). In addition, Zhao et al. (2018) reported that a high dose of BHT could affect the physiological response of *Haematococcus pluvialis*, which significantly decreased biomass production and astaxanthin content (Zhao et al., 2018). In this study, we speculate that 500 μmol L^–1^ of BHT may cause cytotoxic effects on apple fruits, while it needs to be further confirmed. Because fruits treated with 200 μmol L^–1^ of BHT had the least severe decay, we chose 200 μmol L^–1^ of BHT immersion treatment to further investigate the mechanism of BHT-induced resistance against *B. dothidea*. Furthermore, we found that 200 μmol L^–1^ of BHT had no effect on the color and sugar content of apple fruits (data not published). In the previous work, BHT applied by adding to fruit wounds at 50∼1,000 μmol L^–1^ could significantly reduce lesion size, and BHT at 100 μmol L^–1^ was the most effective (Yu et al., 2018). Different methods of applying BHT may account for the inconsistency in these two studies.

The protection of plants from invasion by pathogens is largely dependent on the activation of a highly coordinated defense response. PAL, PPO, GLU, and CHI, the primary defensive enzymes, play important roles in plant defense responses against fungal infection and are widely used as markers to assess induced resistance (Zhang Q. M. et al., 2016; Liu et al., 2019). PAL is a key enzyme in the phenylpropanoid pathway that is involved in the biosynthesis of many compounds related to the structure and resistance of plants, such as lignin and SA, whereas PPO catalyzes the oxidation of phenolic compounds to produce antimicrobial substances (Tian et al., 2006). GLU and CHI encoded by *PR2* and *PR3* can directly inhibit fungal growth through the degradation of fungal cell walls or release oligosaccharide elicitors and induce a consequent chain of defense reactions (Edreva, 2005). Zheng et al. (2011) showed that the activities of these four enzymes in tomato fruits were trigged by L-arginine application, which induced resistance against *B. cinerea*. Zhang Y. et al. (2016) demonstrated that SA treatment increased PAL and PPO activities and up-regulated the expression of *GLU* and *CHI* in apple leaves, resulting in significantly enhanced host resistance against *Glomerella cingulata* infection. Our present study showed that the four defensive enzymes are important components in BHT-treated fruits, which exhibited significant higher enzyme activities than the control (*P* < 0.05). In addition, our results also indicated that BHT treatment could enhance SA levels in apple fruits. These results suggested that BHT could regulate the phenylpropanoid pathway and stimulate the production of antimicrobial substances, which in turn contribute to the induced resistance in apple fruits against *B. dothidea*.

CAT, POD, and SOD are important antioxidant enzymes that regulate the metabolism of ROS and induce resistance in fruits against pathogen invasion (Mittler, 2002). A previous study showed that BHT has significant effect on the activities of these antioxidant enzymes, which is involved in mitigating abiotic stress-induced ROS and metabolite accumulation in *H. pluvialis* (Zhao et al., 2018). In the present study, the activities of CAT, POD, and SOD in BHT-treated fruits were remarkably increased and maintained at higher levels than those in the control fruits during the storage. Correspondingly, in BHT plus *B. dothidea*-treated fruits, the capacity of fruit tissues to scavenge redundant ROS was stimulated, and MDA content remained at a lower level than that in the fruits inoculated with *B. dothidea* alone. These results indicated that high activities of antioxidant enzymes induced by BHT played critical roles in alleviating oxidative stress, which agrees with previous reports (Tareen et al., 2012; Zhang et al., 2018). Additionally, recent research demonstrated that SA treatment could enhance fruit resistance against *B. dothidea* by magnifying the activities of CAT, POD, and SOD (Zhao et al., 2020). Our results also indicated that BHT induced resistance in apple fruits is partially due to the increased activities of antioxidant enzymes.

Salicylic acid, jasmonic acid, and ethylene are critical regulatory signals in plant defense against pathogens, and these signaling pathways are also known to crosstalk in antagonistic or synergistic manners (Mundy et al., 2006). Previous studies showed that SA is involved in the principal pathway in apple fruits to fight against *B. dothidea*, and exogenous SA application could enhance the resistance to *B. dothidea* in apple fruits (Zhao et al., 2020). In our study, *PR1* and *PR5* were strongly induced by BHT in apple fruits, indicating that effective manipulation of SA signaling pathway was essential for fruit resistance conferred by BHT treatment. The transcripts of *PDF1.2* and *COI1*, as well as *CTR1* and *ERF3*, were not induced by BHT, suggesting that JA and ET may be not the main signaling pathways in the BHT-induced resistance. Various studies demonstrated that JA signaling pathway is primarily induced by and effective in resistance against necrotrophic pathogens (Trusov et al., 2006). For example, melatonin induced resistance against *B. cinerea* in tomato fruits by stimulating JA signaling pathway (Liu et al., 2019). Moreover, the synergistic effects of JA and ET signaling pathways are reported to inhibit gray mod in tomato fruits (Yu et al., 2019).

Salicylic acid content and SA synthesis-related gene expression were also determined in this study, which helped us to further determine whether SA signaling pathway mediated the BHT-induced resistance in apply fruits against *B. dothidea*. *SID2* plays an essential role in SA accumulation and plant disease resistance (Dempsey et al., 2011). In apple fruits, *MdSID2* contributed to the production of SA against *B. dothidea* infection (Zhao et al., 2020). *EDS1* and its interacting partner, *PAD4*, constitute a regulatory hub that is essential in the regulation of the plant defense to invasive (hemi-)biotrophic pathogens (Wiermer et al., 2005). Based on our results, the expression levels of *SID2*, *PAD4*, and *EDS1* were up-regulated significantly; and SA content increased remarkably in BHT-treated fruits, which led us to conclude that BHT-induced resistance against apple ring rot was mainly through the accumulation of SA content.

## Conclusion

Butylated hydroxytoluene treatment triggered remarkable disease resistance against apple ring rot caused by *Botryosphaeria dothidea*. BHT could enhance the activities of defensive enzymes CHI, GLU, PAL, and PPO. Meanwhile, it increased antioxidant enzyme activities to alleviate lipid peroxidation. Furthermore, BHT treatment promoted SA content and up-regulated SA defense signaling, which is a crucial pathway in pathogen resistance. These data indicate that BHT confers resistance in apple fruits against *B. dothidea*, possibly by increasing defensive enzyme activities and activating SA signaling pathway. Therefore, our study strongly supports the application of BHT as a new plant activator to inhibit postharvest decay caused by *B. dothidea* in apple fruits.

## Data Availability Statement

The original contributions presented in the study are included in the article/supplementary material, further inquiries can be directed to the corresponding author.

## Author Contributions

YH and CS carried out the experiments and analyzed the data with the help of XG and SL. SL analyzed the data of gene expression and prepared the figure. YH and CW wrote the manuscript with help from all authors. BL performed the manuscript revision and provided part of the financial support. XG reviewed and edited the manuscript. All authors approved its final version.

## Conflict of Interest

The authors declare that the research was conducted in the absence of any commercial or financial relationships that could be construed as a potential conflict of interest.
